# Evaluation of Growth and Development of Late Mixed Dentition Upper Dental Arch with Normal Occlusion Using 3-Dimensional Digital Models

**DOI:** 10.1155/2019/4191848

**Published:** 2019-11-14

**Authors:** Dapeng Yang, Shuran Liang, Ke Zhang, Weimin Gao, Yuxing Bai

**Affiliations:** ^1^The Department of Orthodontics, School of Stomatology, Capital Medical University, Beijing, China; ^2^Department of Orthodontics, Tangshan Union Medical College Hospital, Tangshan, Hebei, China; ^3^Department of Stomatology, Beijing Chao-Yang Hospital, Beijing, China

## Abstract

**Objective:**

The purpose of this study was to observe the three-dimensional growth and development of the maxillary arch in 10-year-olds with normal occlusion during the late mixed dentition stage.

**Methods:**

Forty-four 10-year-old students (22 males and 22 females) who had normal occlusion during late mixed dentition were selected from an elementary school in Beijing, China. Once per year for three consecutive years, a dental cast was obtained from each subject, and the cast was scanned with a 3D digital scanner (R700 3D). The three-dimensional measurements of the maxillary dental arch and the inclination of the bilateral maxillary first molars were obtained from the digital model.

**Results:**

The upper anterior arch length (UAAL), upper total arch length (UTAL), upper inter primary or permanent canine width (UICW), upper intermolar width (UIMW), and upper dental arch length (UDAL) increased by 0.959 mm, 0.583 mm, 0.955 mm, 1.462 mm, and 2.46 mm, respectively, over the two years (*P* < 0.001). UR6BL and UL6BL decreased by 4.416° and 7.133°, respectively, over the two years (*P* < 0.001). The values of the UICW and UIMW were 1.67 mm and 1.86 mm, respectively, larger in males than in females at 12 years old (*P* < 0.01). The change in the UTAL was 0.431 mm greater in males than in females over the 2 years (*P* < 0.05).

**Conclusion:**

The UAAL, UTAL, UICW, UIMW, and UDAL in 10- to 12-year-olds with normal occlusion increased with age. The buccolingual inclination of the bilateral maxillary first molars inclined to the palatal side with age. The UICW and UIMW were larger in males than in females at 12 years old. The male UTAL increased more than the female UTAL over the 2 years.

## 1. Introduction

The three-dimensional (3D) growth and development of the dental arch is a continuous and complex biological process. It includes 3D changes in the width, length, and height of the arch. Because these changes occur at different ages, the measurements for each parameter may vary with age. Moreover, the magnitudes of the changes vary with age. A clear understanding of the 3D changes in the dental arch during each stage of growth and development could be very important for guiding orthodontists in clinical practice.

Many researchers have focused on the growth and development of the dental arch in adults [1–3]. These studies have shown that the growth and development trends of the dental arches in adults are approximately the same. However, many studies have presented controversial or even opposite conclusions about the growth and development of the dental arches of teenagers, especially during the late mixed dentition stage. Ahn et al. [4] performed a follow-up study on a group of 6-year-old children and found that the width between untreated permanent canines decreased. However, Slaj et al. [5] found no significant change in the width between the permanent canines. Regarding arch height, Slaj et al. found that the upper intercanine height decreased during the mixed dentition stage. However, Yang et al. [6] performed a follow-up study on a group of 6-year-old children and found that the upper dental arch height increased. Some studies have reported the mesiodistal and buccolingual inclination of the maxillary first molars [7]. Santana et al. [8] found that the buccolingual inclination of the maxillary molars affected the dental arch width.

In the study of dental arch growth and development, traditional methods are generally based on a dental cast. As technology has advanced, digital models have emerged as useful tools to study the 3D growth and development of the dental arch. The digital model makes it possible to create a reference plane, temporary plane, and grid lines. These make the linear measurements more precise and convenient. The accuracy and reliability of the digital model obtained by applying the 3Shape R700 scanner are reliable, and this scanner can be used to perform orthodontic measurement analyses [9]. Generali et al. [10] evaluated the maxillary dental arch and palate in unilateral cleft lip and palate subjects using 3D laser scanning. The intercanine width of the maxillary dental arch was significantly smaller in patients with unilateral cleft lip and palate than in noncleft lip and palate children. Veli et al. [11] evaluated the dental arch asymmetry in patients with class II subdivision malocclusion with 3-dimensional digital models and found that dental arch asymmetry did not improve or worsen with growth.

The purpose of this study was to use a digital model generated with a 3Shape R700 scanner for 2 years of observations of the 3D growth of the maxillary dental arch and mesiodistal and buccolingual inclinations of the bilateral maxillary first molars. It also investigated whether there was a sex-based difference in the dental arch and dental arch changes.

## 2. Materials and Methods

In this study, 10-year-old students who had normal occlusion during the late mixed dentition stage were selected from an elementary school in Beijing, China. The selection criteria were as follows: Subjects had a class I molar relationship. The contour of the face was symmetrical, with no protrusion or retraction. The full dentition had no caries and or missing teeth. Bilateral primary canines existed, and the mandibular teeth had no interproximal caries. The subjects had no negative oral habits and no history of orthodontic treatment. They had no history of facial trauma or changes in physical condition. Subjects who started orthodontic treatment during the study period were excluded. The study procedure was explained to the subjects and their parents, and written informed consent was obtained from the parents of all the participants before the commencement of the trial.

In total, 44 research subjects were included (22 male and 22 females). All dental casts were scanned with a 3D laser scanner (R7003D Dental Scanner; 3Shape A/S, Copenhagen, Denmark). The models were analyzed by a researcher using 3Shape Orthoanalyzer analysis software (version 10.7.10).

The sagittal plane was drawn on the digital model through two landmarks identified along the median palatal raphe. One landmark was identified as the point on the median palatal raphe adjacent to the second ruga. The other point was identified on the median palatal raphe 1 cm distal to the first point. The coronal plane was 90° to the corresponding sagittal plane and parallel to the distal plane of the upper dental arch model. These two planes were used as reference planes in this study.

**Figure 1:**
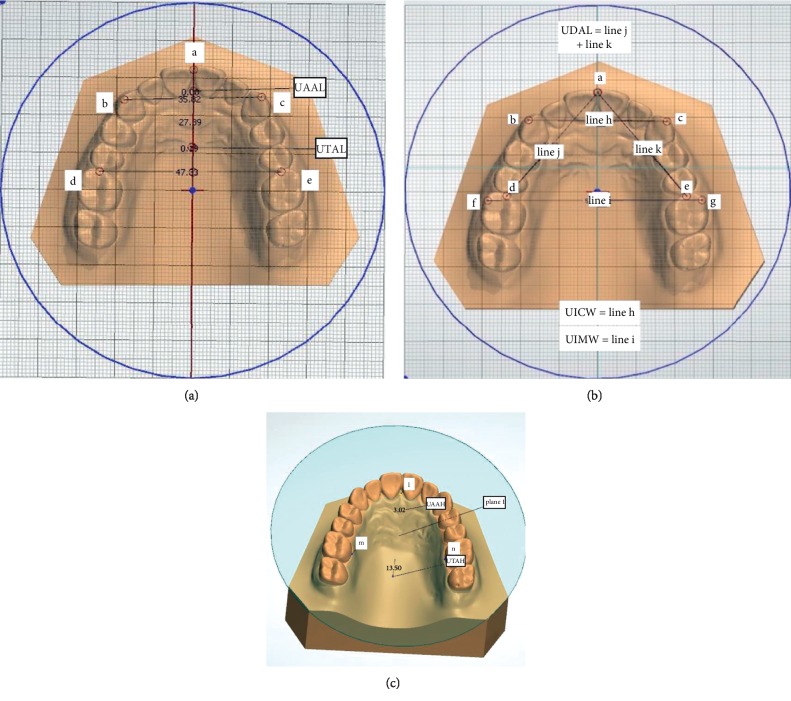
(a) Illustration of UAAL and UTAL. UAAL: connecting points b and c, the vertical distance from point a to the connecting line between point b and point c; UTAL: connecting points d and e, the vertical distance from point a to the connecting line between point d and point e. (b) Illustration of the UICW, UIMW, and UDAL. UICW: connecting points b and c gives line h, which represents the UICW; UIMW: connecting points f and g gives line i, which represents the UIMW; UDAL: connecting points a and d gives line j; and connecting points a and e gives line k. UDAL = line j + line k. (c) Illustration of plane 1, UAAH, and UTAH. Plane 1: established by l, m, and n points; UAAH: the distance from the highest point toward the canine on the midline palatine suture to plane 1; UTAH: the distance from the highest point toward the maxillary first molar on the midline palatine suture to plane 1.

**Figure 2:**
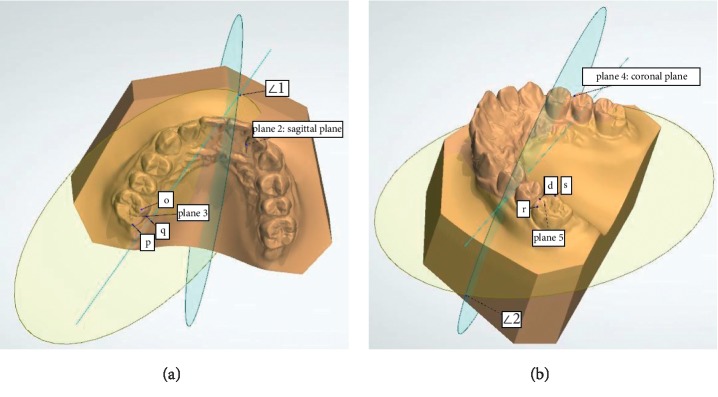
(a) Illustration of plane 2, plane 3, and ∠1. Plane 2: sagittal plane (blue); plane 3: established by o, p, and q points (yellow); and ∠1: angle of buccolingual inclination, established by plane 2 and plane 3. (b) Illustration of plane 4, plane 5, and ∠2. Plane 4: coronal plane (blue); plane 5: established by r, s, and d points; ∠2: angle of mesiodistal inclination, established by plane 4 and plane 5.

The definitions of the landmarks and measurements of dental arch dimensions are shown in Table 1 and Figures 1 and 2.

In the 3D model of the growth and development of the dental arch, we defined a new plane (plane 1) and two new angles (∠1 and ∠2). In the measurement of the upper dental arch height on a conventional dental cast, the reference plane is the occlusion plane of the upper dentition, and thus, the measurement can be affected by the growth of the tooth crown. The new reference plane (plane 1) was created through three points, as shown in Table 1 and Figure 1(c). The establishment of plane 1 avoids the impact of the upper tooth crown growth.

### 2.1. Method Error

SPSS software was used for the statistical analysis. Twenty-two subjects were randomly selected and measured again by the same operator 2 weeks after the initial measurement. To calculate the measurement error, Dahlberg's formula was applied. One-way ANOVA followed by Tukey's post hoc test and the *t*-test were applied to identify any statistically significant differences between males and females for each measurement. The level of significance was set at *P* < 0.05.

## 3. Results

**Figure 3:**
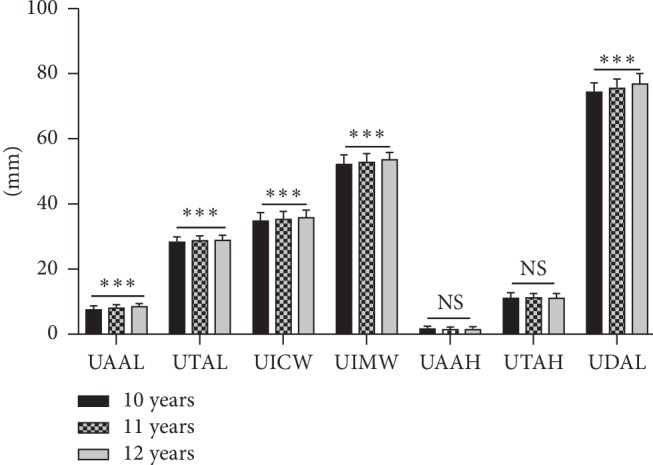
Comparison of upper dental arch dimensions at different ages. NS, not significant; ^*∗*^*P* < 0.05, ^*∗∗*^*P* < 0.01, ^*∗∗∗*^*P* < 0.001.

**Figure 4:**
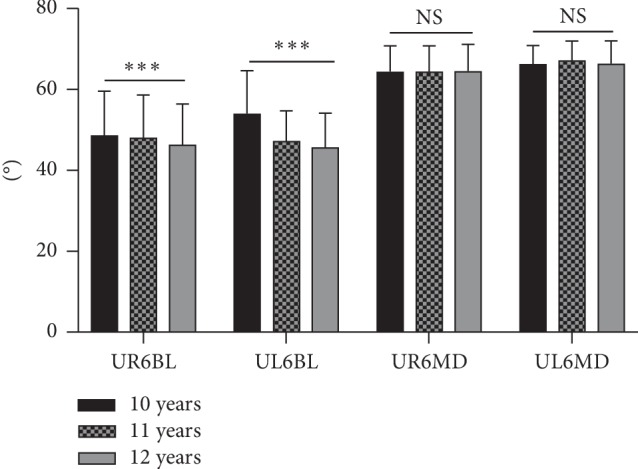
Comparison of buccolingual and mesiodistal inclinations (°) of the upper first molar at different ages. NS, not significant; ^*∗*^*P* < 0.05, ^*∗∗*^*P* < 0.01, ^*∗∗∗*^*P* < 0.001.

**Figure 5:**
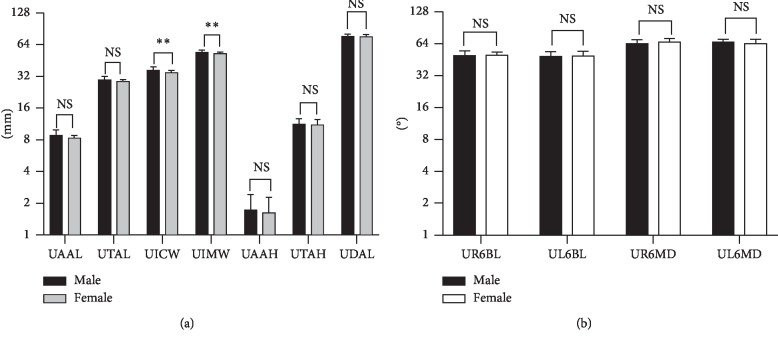
(a) Comparison of upper dental arch dimensions between males and females at 12 years old. (b) Comparison of buccolingual and mesiodistal inclinations (°) of the upper first molar between males and females at 12 years old. NS, not significant; ^*∗*^*P* < 0.05, ^*∗∗*^*P* < 0.01, ^*∗∗∗*^*P* < 0.001.

**Figure 6:**
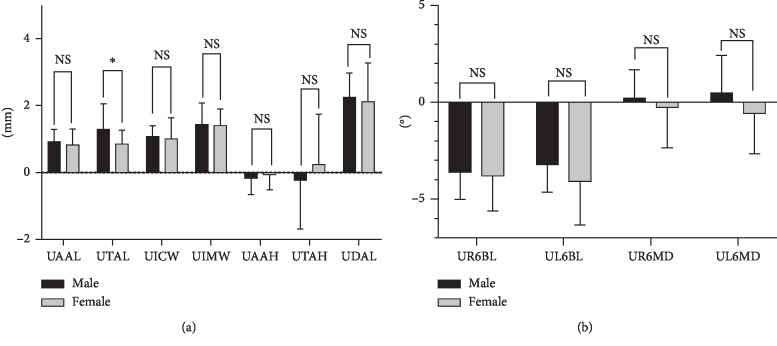
(a) Comparison of changes in the upper dental arch dimensions over the 2 years between males and females. (b) Comparison of changes in the buccolingual and mesiodistal inclinations of the upper first molar over the 2 years between males and females. NS, not significant; ^*∗*^*P* < 0.05, ^*∗∗*^*P* < 0.01, ^*∗∗∗*^*P* < 0.001.

The means and standard deviations of the upper dental arch dimensions at different ages are shown in Table 2 and Figure 3. The values for UAAL, UTAL, UICW, UIMW, and UDAL increased 0.959 mm, 0.583 mm, 0.955 mm, 1.462 mm, and 2.46 mm, respectively, over the past two years (*P* < 0.001). The means and standard deviations of the buccolingual and mesiodistal inclinations of the upper first molar at different ages are shown in Table 3 and Figure 4. UR6BL and UL6BL decreased 4.416° and 7.133°, respectively, over the two years (*P* < 0.001). The comparison of the upper dental arch dimension between 12-year-old males and females is shown in Table 4 and Figure 5(a). The values of UICW and UIMW were 1.67 mm and 1.86 mm, respectively, larger in males than in females at 12 years old (*P* < 0.01). The comparison of the changes in the upper dental arch dimensions over the 2 years between males and females is shown in Table 5 and Figure 6(a). In addition, the change in the UTAL was 0.431 mm greater in males than in females over the 2 years (*P* < 0.05).

## 4. Discussion

A 2-year-follow-up of 10-year-olds with normal occlusion was performed in this study, and the UAAL and UTAL increased significantly with age. This is consistent with the results of the Louly et al. [12] and Alkadhi et al. [13] studies. The UTAL is the perpendicular distance from the mesial contact point of the central incisor to the connecting line between the mesial contact points of the bilateral maxillary first molars. According to the definition, a change in UTAL is not only affected by the growth and development of the maxillary arch but also by mesiodistal movement and the inclination of the maxillary first molar, which affects the position of the mesial contact point. However, the UTAL value in this study was less influenced by the mesiodistal inclination of the maxillary first molar than in previous studies. Moreover, there was no statistically significant change in the mesiodistal inclination of the maxillary first molar, which further reduced the effect of this factor on UTAL. There was no gender-based difference in UAAL and UTAL at 12 years old. The result is consistent with the finding of Okori et al. [14]. The change in the UTAL was larger in males than in females over the 2 years.

Corroborating with the findings of previous studies [15, 16], the UICW and UIMW increased with age over the two years, and the values of UICW and UIMW were larger in males than in females at 12 years old. However, some studies have shown that the UIMW decreases over time [17]. As seen in Figure 1(b), the measurement of the UIMW involves the position of its cusp. The position of the cusp of the maxillary molar can be affected by the buccolingual inclination of the maxillary first molar. This current study has shown that the buccolingual inclination of the maxillary first molar becomes inclined to the palatal side with age. This is consistent with the findings of Sayania et al. [18]. However, the UIMW did not decrease due to the palatal inclination of the maxillary molars; rather, it significantly increased with age. This may be because, the growth of the maxillary dental arch leads to an increase in the width of the maxillary alveolar bone and UIMW [19], which offsets the reduction in the UIMW caused by the palatal inclination of the maxillary first molar. The findings of this study are consistent with the findings of Santana et al. [8]. Due to the increase in the width of the maxillary dental arch at this stage, some patients with mild to moderate crowding are not encouraged to undergo tooth extraction immediately in clinical practice. It is reasonable to observe the amount of growth of the dental arch in the horizontal direction or to perform maxillary expansion to solve the problem. In terms of the timing of maxillary expansion, the study by Baccetti et al. [20] suggested that patients undergoing rapid maxillary expansion before the peak of growth and development could have more effective long-term changes in the maxilla and maxillary structures at the bone level.

For the measurement of the height of the maxillary arch, we chose a landmark point to eliminate the measurement error caused by the eruption of the teeth. Regarding the dental arch height, this study showed that UTAH and UAAH did not change significantly with age over the two-year period. In contrast, previous studies [21] have shown that the height of the arch decreases with age. In clinical practice, for patients with high palates, we do not recommend performing maxillary expansion immediately. The decline of the palate and the decrease in the vertical growth rate of the alveolar process may solve this problem spontaneously. In patients with deep overbites, the slowing of the vertical growth rate of the alveolar process and the eruption of the posterior teeth may also reduce the deep overbite or even render it normal.

In this study, the UDAL increased with age. This is contrary to the results of previous studies [22]. The definition of the UDAL used in this study is shown in Figure 1(b). According to this definition, the UDAL measurement was less affected by the mesiodistal and buccolingual inclination in the current study than in the previous studies. The study by Marshall et al. [23] on the buccolingual tilting of the crown of the molars showed that the maxillary molars had a buccal torque when erupted; however, at later stages, the maxillary first molars showed a tendency to incline toward the palatal side. Therefore, the previous studies that were greatly affected by the inclination of the maxillary molars have shown that the UDAL decreases with age. Consequently, the conclusion drawn in this study was contrary to the conclusions drawn in previous studies.

Sayania et al. [18] longitudinally studied the buccolingual inclination of the first molar and showed that the maxillary first molar had a buccal inclination when erupted and that it stood upright with increasing age. Yang and Chung [7] compared the buccolingual inclination of the molars of untreated adults and children. The conclusion drawn was that the maxillary first molars exhibited buccal inclination, and the inclination in adults is more palatal than that in children. In this study, the maxillary bilateral first molars exhibited palatal inclination over two years, while the mesiodistal inclination did not change significantly, and there was no gender-based difference. This finding is consistent with the conclusions of the aforementioned studies. Because this study had a small sample size and the subjects had class I skeletal malocclusions, further studies with larger sample sizes and different skeletal malocclusions in the study population should be considered to observe changes in the buccolingual and mesiodistal inclinations of the first maxillary molars bilaterally with age, thereby providing clinical reference for orthodontists.

## 5. Conclusion

1. The UAAL, UTAL, UICW, UIMW, and UDAL in 10- to 12-year-olds with normal occlusion increased with age. The buccolingual inclination of the bilateral maxillary first molars inclined to the palatal side with age. The mesiodistal inclination of the bilateral maxillary first molars did not change with age.
2. The UICW and UIMW were larger in males than in females at 12 years old.
3. The UTAL increased more in males than in females over the 2 years.

## Figures and Tables

**Table 1:** Landmarks and measurements of dental arch dimensions.

Landmark and acronym	Description
a	The midpoint between the proximal tip of the upper central incisors
b	Upper right primary or permanent canine cusp tip
c	Upper left primary or permanent canine cusp tip
d	Mesial anatomic contact point of the right upper first molar
e	Mesial anatomic contact point of the left upper first molar
f	Upper right first molar mesiobuccal cusp tip
g	Upper left first molar mesiobuccal cusp tip
l	Most convex point of the incisal papillae
m	Most lingual point on the gingival margin of the lingual surface of permanent upper first right molar
n	Most lingual point on the gingival margin of the lingual surface of permanent upper first left molar
o	Upper right first molar mesial lingual cusp tip
p	Upper right first molar distal lingual cusp tip
q	Most gingival point at the lingual groove of the maxillary first permanent molars
r	Upper first molar mesiobuccal cusp tip
s	Upper first molar mesiolingual cusp tip
UAAL	Upper anterior arch length
UTAL	Upper total arch length
UICW	Upper inter primary or permanent canine width
UIMW	Upper intermolar width
UDAL	Upper dental arch length
UAAH	Upper anterior arch height
UTAH	Upper total arch height
UR6BL	Buccolingual inclination of the upper right first molar
UL6BL	Buccolingual inclination of the upper left first molar
UR6MD	Mesiodistal inclination of the upper right first molar
UL6MD	Mesiodistal inclination of the upper left first molar

**Table 2:** Means and standard deviations of upper dental arch dimensions at different ages.

Measurement (mm)	10 years (*n* = 44)		11 years (*n* = 44)		12 years (*n* = 44)		*P*
	Mean	SD	Mean	SD	Mean	SD	
UAAL	7.711	1.040	8.279	0.847	8.670	0.778	^*∗∗∗*^
UTAL	28.500	1.425	28.882	1.301	29.083	1.357	^*∗∗∗*^
UICW	35.013	2.356	35.456	2.234	35.968	2.140	^*∗∗∗*^
UIMW	52.355	2.695	53.036	2.437	53.817	2.051	^*∗∗∗*^
UAAH	1.817	0.649	1.730	0.612	1.691	0.653	NS
UTAH	11.459	1.503	11.382	1.174	11.265	1.289	NS
UDAL	74.621	2.606	75.703	2.708	77.081	2.300	^*∗∗∗*^

*n*, number; SD, standard deviation; NS, not significant; ^*∗*^*P* < 0.05, ^*∗∗*^*P* < 0.01, ^*∗∗∗*^*P* < 0.001.

**Table 3:** Means and standard deviations of buccolingual and mesiodistal inclinations of the upper first molar at different ages.

Measurement (°)	10 years (*n* = 44)		11 years (*n* = 44)		12 years (*n* = 44)		*P*
	Mean	SD	Mean	SD	Mean	SD	
UR6BL	50.528	6.982	49.168	7.000	47.161	7.098	^*∗∗∗*^
UL6BL	51.789	6.434	47.944	5.970	46.665	7.241	^*∗∗∗*^
UR6MD	64.403	6.370	64.299	6.509	64.573	6.589	NS
UL6MD	66.317	4.508	67.077	4.909	65.369	5.660	NS

*n*, number; SD, standard deviation; NS, not significant; ^*∗*^*P* < 0.05, ^*∗∗*^*P* < 0.01, ^*∗∗∗*^*P* < 0.001.

**Table 4:** Comparison of upper dental arch dimensions between males and females at 12 years old.

Measurement (mm)	Age (years)	Male (*n* = 22)		Female (*n* = 22)		*P*
		Mean	SD	Mean	SD	
UAAL	12	8.786	0.366	8.419	0.223	NS
UTAL	12	29.660	0.750	28.910	0.443	NS
UICW	12	36.660	1.664	34.990	0.610	^*∗∗*^
UIMW	12	54.750	1.857	52.890	0.556	^*∗∗*^
UAAH	12	1.741	0.040	1.700	0.200	NS
UTAH	12	11.330	0.130	11.200	0.393	NS
UDAL	12	77.610	1.049	76.570	0.902	NS

*n*, number; SD, standard deviation; NS, not significant; ^*∗*^*P* < 0.05, ^*∗∗*^*P* < 0.01, ^*∗∗∗*^*P* < 0.001.

**Table 5:** Comparison of changes in the upper dental arch dimensions over 2 years between males and females.

Measurement (mm)	Male (*n* = 22)		Female (*n* = 22)		*P*
	Mean	SD	Mean	SD	
UAAL	0.937	0.358	0.833	0.465	NS
UTAL	1.296	0.758	0.865	0.402	^*∗*^
UICW	1.082	0.321	1.016	0.622	NS
UIMW	1.441	0.639	1.417	0.489	NS
UAAH	0.177	0.480	0.075	0.438	NS
UTAH	0.239	1.449	0.251	0.494	NS
UDAL	2.254	0.722	2.126	1.154	NS

*n*, number; SD, standard deviation; NS, not significant; ^*∗*^*P* < 0.05, ^*∗∗*^*P* < 0.01, ^*∗∗∗*^*P* < 0.001.

## Data Availability

The data used to support the findings of this study are available from the corresponding author upon request.
